# DNA Gyrase Inhibitors Increase the Frequency of Bacteriophage-like RcGTA-Mediated Gene Transfer in *Rhodobacter capsulatus*

**DOI:** 10.3390/genes13112071

**Published:** 2022-11-09

**Authors:** Rachel Bernelot-Moens, J. Thomas Beatty

**Affiliations:** Department of Microbiology and Immunology, The University of British Columbia, 2350 Health Sciences Mall, Vancouver, BC V6T 1Z3, Canada

**Keywords:** *Rhodobacter*, gene transfer agent, RcGTA, horizontal gene transfer, phage, virus, DNA gyrase, novobiocin, ciprofloxacin, antibiotic, subinhibitory concentration

## Abstract

*Rhodobacter capsulatus* produces a bacteriophage-like particle called the gene transfer agent (RcGTA) that mediates horizontal gene transfer. RcGTA particles transfer random ~4.5-kb fragments of genomic DNA that integrate into recipient genomes by allelic replacement. This work addresses the effect of sub-inhibitory concentrations of antibiotics on gene transfer by RcGTA. A transduction assay was developed to test the effects of various substances on gene transfer. Using this assay, low concentrations of DNA gyrase inhibitors were found to increase the frequency of gene transfer. Novobiocin was studied in more detail, and it was found that this antibiotic did not influence the production or release of RcGTA but instead appeared to act on the recipient cells. The target of novobiocin in other species has been shown to be the GyrB subunit of DNA gyrase (a heterotetramer of 2GyrA and 2GyrB). *R. capsulatus* encodes GyrA and GyrB homologues, and a GyrB overexpression plasmid was created and found to confer resistance to novobiocin. The presence of the overexpression plasmid in recipient cells greatly diminished the novobiocin-mediated increase in gene transfer, confirming that this effect is due to the binding of novobiocin by GyrB. The results of this work show that antibiotics affect gene transfer in *R. capsulatus* and may be relevant to microbial genetic exchange in natural ecosystems.

## 1. Introduction

*Rhodobacter capsulatus* is a metabolically versatile α-proteobacterium that produces a gene transfer agent (RcGTA), a virus-like element with the only known function of mediating horizontal gene transfer (HGT). Several species of bacteria from the order *Rhodobacterales* produce homologous GTAs, and genes encoding RcGTA-like GTAs are conserved in most genomes in the *Rhodobacterales* as well as many genomes of species in other alphaproteobacterial orders [1].

Gene transfer agents (GTAs) as a class carry out HGT using a mechanism resembling generalized transduction, in which random or quasi-random genomic DNA fragments are packaged into particles resembling small bacteriophages, which are released into the environment and introduce genes into other cells [1,2,3].

The genes encoding RcGTA and regulatory proteins are scattered over the *R. capsulatus* chromosome in 5 locations [4,5,6]. Production and release of RcGTA particles occurs in a small subset (<3%) of cells in a population [5,7], and the frequency is controlled by an extracellular repressor [8].

The interaction of RcGTA with recipient cells begins with binding to a polysaccharide capsule, followed by injection of DNA into the periplasm. DNA is translocated into the cytoplasm by homologues of competence (natural transformation) proteins, and incorporation of genes into the genome requires proteins involved in homologous recombination [9,10,11,12].

Subinhibitory concentrations of antibiotics (concentrations that allow growth to occur at the same or similar rate of growth as in the absence of antibiotic) have been shown to have profound effects on bacterial transcription profiles [13]. The SOS response and HGT by transformation [14], conjugation [15], and phage-mediated transduction [16] may be stimulated by subinhibitory concentrations of antibiotics. Additionally, the spirochaete *Brachyspira hyodysenteriae* contains a GTA (not homologous to RcGTA) called VSH-1 that is induced by subinhibitory concentrations of the antibiotics carbadox and metronidazole [1,17]. Here, we report the effects of subinhibitory concentrations of antibiotics on RcGTA-mediated genetic exchange.

We developed an assay to investigate the effect of substances on the frequency of gene transfer, and applied this assay to subinhibitory concentrations of antibiotics. The antibiotics we found to have the greatest effect on gene transfer are DNA gyrase inhibitors, the aminocoumarins novobiocin (Nb) and clorobiocin (Cb), and the fluoroquinolone ciprofloxacin (Cip). DNA gyrase is an essential bacterial enzyme that functions in altering DNA supercoiling stresses during DNA replication and transcription [18]. DNA gyrase inhibitors usually act via two mechanisms. Either the enzymatic activity of gyrase is inhibited or the covalent enzyme-DNA complex is stabilized. Aminocoumarins inhibit the ATPase activity of gyrase by acting on the B subunit (GyrB), whereas fluoroquinolones trap gyrase-cleaved DNA in a subunit A/DNA cleavage complex [19,20,21].

Because Nb, Cb, and Cip all caused an increase in RcGTA-mediated gene transfer, it appears that the effect is due to the overall inhibition of DNA gyrase activity, as opposed to the consequence of a particular mechanism. Interestingly, this increase in gene transfer was not due to increased production of RcGTA, but instead appeared to be due to the inhibition of DNA gyrase in recipient cells. We suggest that the frequency of allelic recombination is increased in recipient cells as a result of a change in DNA supercoiling that affects gene expression in *R. capsulatus*, resulting in changes in the amounts of proteins needed for import and integration of RcGTA-borne genes.

## 2. Materials and Methods

### 2.1. Bacterial Strains and Growth Conditions

The *E. coli* strain DH5α was used for cloning, and the strains S17-1 and C600 (pDPT51) were used to conjugate plasmids into *R. capsulatus*. *E. coli* strains were grown in lysogeny broth (LB) medium [22] supplemented with antibiotics as needed at the following working concentrations (in µg/mL): ampicillin, 150; kanamycin, 50; spectinomycin, 100; gentamicin, 10.

The *R. capsulatus* strains used are described in Table 1. Strain ∆RC6 contains a 1.6-kb *npt*II segment of Tn5 (encoding kanamycin resistance) in place of photosynthesis genes (*pufLMX*) needed for phototrophic growth. Strain DW5 contains a 701 bp deletion of a third reaction center gene (*puhA*) also needed for phototrophic growth. *R. capsulatus* cultures were grown chemotrophically or phototrophically in RCV minimal medium [23] or YPS complex medium [24], supplemented with appropriate antibiotics at the following working concentrations (in µg/mL): kanamycin, 10; spectinomycin, 10; gentamicin, 5; rifampicin, 80.

Culture density was monitored by measuring optical density at 660 nm or by measuring light scattering with a Klett-Summerson photometer (red filter #66). An OD_660_ of 1.0 is approximately equal to 130 Klett units (KU), and 4.8 × 10^8^ cfu/mL.

### 2.2. Recombinant DNA Techniques, Plasmids, and Primers

Standard methods of DNA isolation, analysis, modification and cloning were used [22].

### 2.3. Transduction Assay

Non-phototrophic strains DW5 and ∆RC6 were grown separately with aeration overnight in YPS medium, harvested by centrifugation, and suspended in 10 mL YPS at a concentration of 20 KU either alone as controls, or mixed in a 1:1 ratio. After incubation with aeration at 30 °C until the cultures had entered the stationary phase, equal numbers of cells (about 10^8^ to 10^9^ cells, based on turbidity) were plated onto YPS agar plates in 3 mL of molten (at ~47 °C YPS 0.4% agar, and recipients of wild type photosynthesis gene alleles selected by acquisition of phototrophic growth capability, using incubation in anaerobic jars with illumination for 2 to 3 days (see Figure S1).

### 2.4. RcGTA Bioassay

Bioassays were performed as described [30] with slight modifications. Donor and recipient cultures were grown aerobically at 30 °C in YPS medium until reaching the stationary phase. Filtered (0.2 µm pore diameter), cell-free supernatant (100 µL) from donor cultures was incubated for one h at 30 °C with 100 µL of recipient cells suspended in 500 µL G buffer (10 mM Tris-HCl (pH 7.8), 1 mM MgCl_2_, 1 mM CaCl_2_, 1 mM NaCl, 500 mg/mL bovine serum albumin). The mixture was spread over YPS agar plates in 3 mL of molten YPS soft agar (0.4%, at ~47 °C) and incubated for four h at 30 °C to allow for expression of newly acquired genes. Gene transfer recipients were selected by acquisition of phototrophic growth capability or antibiotic resistance (see Figure S1).

### 2.5. SDS-PAGE and Western Blot

*R. capsulatus* cultures were grown for 24 h from a starting density of 20 KU and cellular and extracellular samples were separated by centrifugation. Cell-free culture supernatant was concentrated by lyophilization. Portions of cellular and extracellular samples from equivalent culture volumes at the same cell concentration, determined by culture absorbance at 660 nm, were boiled for 10 min in sample loading buffer (50 mM Tris-HCl pH 6.8, 2% SDS, 10% glycerol, 0.1% bromophenol blue, 1% *β*-mercaptoethanol), separated on 12% SDS-PAGE gels and blotted onto nitrocellulose membranes. Blotting was performed with a Mini Trans-Blot apparatus (BioRad) in Electroblot Buffer (27.5 mM Tris-Base, 192 mM glycine, 20% methanol) at 100 V for 1.5 h. Blots were probed with a rabbit primary antibody raised against the *R. capsulatus* RcGTA capsid protein [31], followed by donkey anti-rabbit Ig secondary antibody linked to peroxidase (Amersham). Protein bands were detected using an electrochemiluminescence (ECL) kit according to manufacturer’s instructions (Amersham), and exposure to X-ray film.

### 2.6. Disruption of RcGTA Gene g4

The spectinomycin resistance-encoding omega fragment (Ω) was excised from plasmid pHP45 [32] by SmaI digestion and inserted into the EcoRV site of the g4 (prohead protease) gene of cosmid p9H54 [33]. The resultant construct (p9H54Ω) was recombined in vivo with plasmid pDPT51 [34] in *E. coli* strain C600, and conjugated into the RcGTA overproducer strain Y262 to obtain RcGTA that was used to transduce the recipient strain, ΔRC6. Recipients with a disrupted g4 gene were selected for by spectinomycin resistance. The DW5 g4 disruption was created in a similar manner, except the g4 gene in p9H54 was disrupted with a KIXX cartridge [35], and the mutants were selected by acquisition of kanamycin resistance. Inability to produce RcGTA in both mutants was confirmed by lack of mature capsid protein in a Western blot.

### 2.7. Conjugation

Stationary phase *E. coli* donor cells were grown overnight in LB supplemented with the appropriate antibiotic, whereas stationary phase *R. capsulatus* recipient cells were grown overnight in RCV medium. Donor cells (200 µL) were harvested by centrifugation, washed and suspended in RCV medium, and then mixed with 500 µL of recipient culture. The mixture was spotted onto a 0.2 µm filter on an RCV agar plate and incubated at 30 °C overnight. The cells in this mixture were suspended and plated on RCV with selection for resistance to the appropriate antibiotic.

### 2.8. Construction of gyrB Overexpression Plasmid

The *R. capsulatus* SB1003 *gyrB* (rcc00004) coding region was amplified by PCR and ligated into the expression vector pRhokHi-6 [36] between the BamHI and NdeI restriction sites. This yielded plasmid pRhoKGyrB, in which the *gyrB* coding region is transcribed from the constitutive *aphII* promoter, and the mRNA translated using the ribosome-binding site provided by the plasmid, producing a C-terminally 6 His-tagged protein.

### 2.9. Statistical Analysis

Statistical significance was determined using the unpaired, two-tailed student’s *t*-test.

## 3. Results

### 3.1. Development of the Transduction Assay

**Figure 1:**
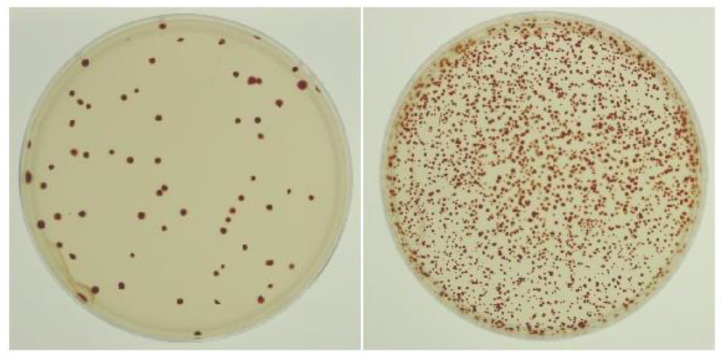
Representative plates from a transduction assay. (**Left**) mixed culture of strains DW5 and ∆RC6 grown in the absence of antibiotic. (**Right**) mixed culture of strains DW5 and ∆RC6 grown in the presence of Nb at 2 µg/mL, showing an estimated 30-fold increase in the number of colonies, and therefore gene transfer. Selection was for phototrophic growth, and each strain grown and plated alone yielded no colonies whatsoever.

This assay to examine the frequency of gene transfer between two strains is based on two non-phototrophic mutants: strain DW5 (containing a translationally in-frame deletion of the reaction center *puhA* gene) and ∆RC6 (containing a kanamycin-resistance cartridge in place of the reaction center *pufLMX* genes), which were cultivated together in mixed culture. Gene transfer could occur in either direction, and restoration of the ability to grow phototrophically was used as an indicator of gene transfer. After plating the mixture on an agar medium and selecting for phototrophic growth, the number of colonies yielded a quantitative measurement of the frequency of gene transfer events. Figure 1 shows an example of the appearance of plates in which there was a great difference between the treated and untreated cultures. In this assay, the effect of any substance on gene transfer can be determined by adding it to the mixture of cultures, incubating, and comparing the number of photosynthetic colonies obtained with that of a control with no substance added.

### 3.2. DNA Gyrase Inhibitors Increase the Frequency of RcGTA-Mediated Gene Transfer

**Figure 2:**
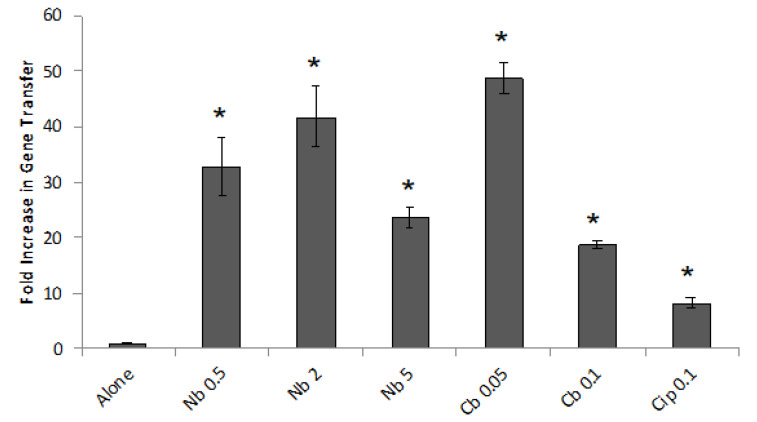
Results of transduction assays evaluating the effects of DNA gyrase inhibitors. Cultures (mixtures of equal numbers of strains DW5 and ∆RC6) were grown in triplicate with or without antibiotics. On the horizontal axis: Nb, novobiocin; Cb, clorobiocin; Cip, ciprofloxacin; numbers indicate the concentrations in µg/mL. The vertical axis gives the frequency of gene transfer relative to the average of cultures grown in the absence of an antibiotic (alone). The asterisk * indicates *p* < 0.01 in pairwise comparisons of cultures grown in the presence and absence of an antibiotic. Error bars show the standard deviation, n = 3.

Several types of antibiotic and other substances were screened and found to have little or no effect on gene transfer (Table S1). However, low concentrations of Cip (a DNA gyrase subunit A inhibitor) and inhibitors of gyrase subunit B (Nb and Cb) were found to cause relatively large increases in the frequency of gene transfer in the transduction assay (Figure 2). In subsequent work we focused on the effect of Nb, and the absence of *R. capsulatus* growth inhibition of 0.5 and 2.0 µg/mL concentrations is shown in Figure S2.

The direction of gene transfer was tested by patching colonies onto YPS agar plates containing rifampicin that were incubated with selection for phototrophic growth. Only DW5 contains the rifampicin resistance gene, and so only those colonies that were a result of DW5 receiving a photosynthesis gene from ∆RC6 would be able to grow phototrophically in the presence of rifampicin. It was unlikely that a rifampicin resistant colony could have arisen due to RcGTA-mediated transfer of both the required photosynthesis gene and the rifampicin resistance gene from DW5 to ∆RC6, because the probability of a double transfer event is extremely low. Although individual RcGTA gene transfers may occur independently with a frequency as high as 10^−5^, the frequency of two such events occurring simultaneously would be 10^−10^ [37]. All transductants examined (n = 90) were resistant to rifampicin, showing that transfer occurred from ∆RC6 to DW5. It appears that the packaging frequency of the smaller *puhA* wild type allele from a ∆RC6 donor was much greater than of the larger *pufLMX* wild type alleles from a DW5 donor, evidently due to the RcGTA head maximal capacity for linear DNA of ~4.5 kb in length [38]. The length of chromosomal DNA deleted from the *puhA* gene in strain DW5 is about 710 bp [28], whereas the length of the *pufLMX* deletion in ∆RC6 is about 4.5 kb [27].

The transduction assay was also performed using the strain ΔLHII (which contains a spectinomycin resistance cartridge inserted into the non-essential *puc* (LHII) operon as well as the rifampicin resistance gene from the SB1003 parent strain) instead of DW5, and recombinants were selected by the presence of both kanamycin resistance (from ΔRC6) and rifampicin resistance (from ΔLHII) in chemotrophic growth, instead of phototrophic growth (Figure S3). The direction of transfer was determined by testing the doubly resistant transductants for spectinomycin resistance, because only with transfer of kanamycin resistance from ΔRC6 to ΔLHII would a cell be resistant to all three antibiotics. It was found that transfer between these two strains occurred in both directions, although mostly from ΔLHII to ΔRC6 (4 of 52 transductants tested were resistant to all three antibiotics). Evidently the frequency of transfer of rifampicin resistance (a point mutation) was greater than the transfer frequency of the ~1.2 kb-long kanamycin resistance cartridge (see Discussion section).

The possibility that an RcGTA-independent gene transfer mechanism was operating was evaluated by disruption of the RcGTA gene g4 (rcc01686) encoding a protease needed for capsid maturation [33]. Disruption of this gene in both strains DW5_Δg4 and ΔRC6_Δg4 used in a transduction assay resulted in the absence of recombinants. Therefore, this assay is not only sensitive, but also specific for RcGTA-mediated gene transduction.

**Figure 3:**
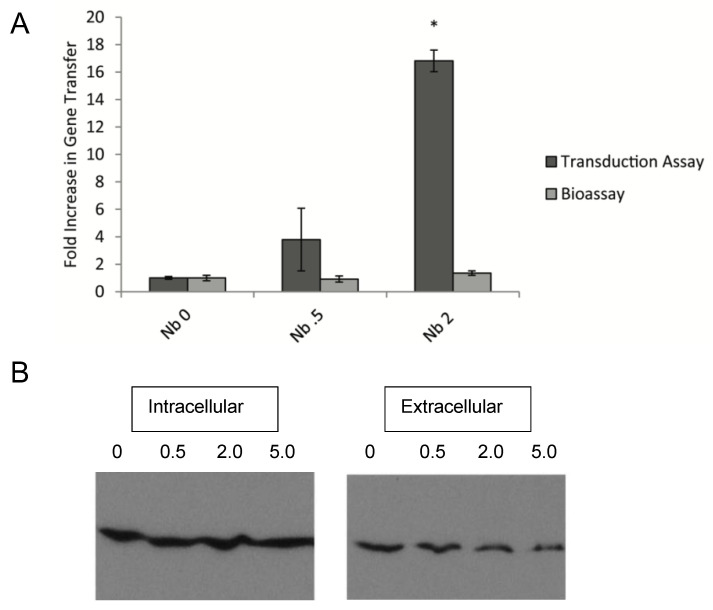
Comparison of gene transfer in a transduction assay and bioassay, and RcGTA levels, in response to the presence of Nb. (**A**) Transduction assay cultures (mixture of strains DW5 and ∆RC6) were grown in duplicate and used for transduction assays as well as for supernatant donors in bioassays. The numbers below the graph give the concentration of Nb in ug/mL in the cultures. * *p* < 0.05 compared to cultures grown without antibiotic. Error bars show the range. (**B**) Cells (intracellular) and cell-free supernatants (extracellular) from transduction assay cultures were examined by Western blot, probed with RcGTA capsid antiserum. The numbers above each lane give the concentration of Nb in ug/mL in the cultures.

The effects of Nb on production and release of RcGTA were investigated further using a bioassay, in which RcGTA produced during the co-culture of DW5 and ∆RC6 in the presence or absence of Nb was separated from cells, and used to transfer phototrophic growth capability to strain DW5. Despite an increase in gene transfer in the presence of Nb in the transduction assay, no increase in the number of phototrophic colonies when RcGTA was obtained from cultures grown in the presence of Nb, compared to cultures grown in the absence of antibiotic (Figure 3A). This indicates that there was no difference in the amount of RcGTA present in cell-free supernatants of cultures grown in the presence or absence of Nb.

When cultures were analyzed by Western blot, Nb did not reproducibly affect the levels of RcGTA (Figure 3B and Figure S4), consistent with the frequencies of gene transfer in the bioassays. Therefore, we hypothesized that Nb had little effect on the production of RcGTA, but instead greatly increased the capability of cells to obtain new alleles from RcGTA particles.

### 3.3. Kinetics of the Novobiocin Effect in the Transduction Assay

**Figure 4:**
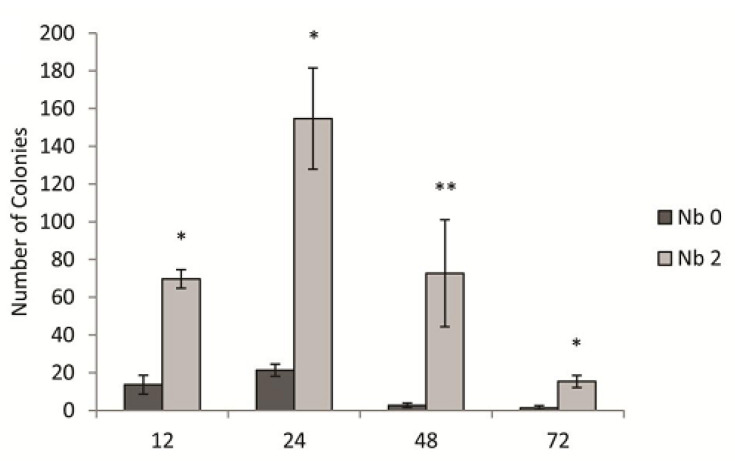
Time course of transduction assays in the presence or absence of 2 µg/mL novobiocin (Nb). Transduction assay of strains ∆RC6 and ∆LHII with time points at 12, 24, 48 and 72 h, yielding 5.1-, 7.3-, 27.3-, and 11.5-fold average increases in gene transfer, respectively, compared to cultures grown without Nb at the same time point. Error bars give standard deviations (n = 3), and * *p* < 0.05, ** *p* < 0.1 relative to the control cultures grown without Nb at each time point.

Because of the variability of the fold-increase in gene transfer due to Nb in the transduction assay (compare 2 µg/mL fold-increases in Figure 1, Figure 2 and Figure 3), we hypothesized that the frequency of transduction in the Nb-treated and/or untreated cultures varies significantly depending on exactly how long the cultures were grown into the stationary phase. As shown in Figure 4, there are indeed substantial changes in the transduction frequency in both the Nb-treated and untreated cultures over the 72 h time period of these experiments. As a result, the increase in transduction due to Nb varied from 5.1 to 27.3-fold. Nevertheless, these experiments confirm that the Nb effect is consistently substantial over a wide range of time scales, and we suggest that variations in the exact degree of increase in transduction frequency do not affect our general conclusion that subinhibitory concentrations of Nb stimulate RcGTA-mediated gene transfer.

### 3.4. Overexpression of gyrB Increases Resistance to Novobiocin and Inhibits the Novobiocin-Dependent Increase in Gene Transfer

Because Nb has been shown to bind to the GyrB protein in other species [19], and *R. capsulatus* contains homologues of *gyrA* and *gyrB* genes, we hypothesized that the *R. capsulatus* GyrB protein is the target of Nb-stimulation of RcGTA-mediated gene transfer.

To address whether Nb-binding to GyrB stimulates gene transfer, we over-expressed the *R. capsulatus gyrB* homologue (*rcc00004*) in strains DW5 and ∆RC6, using the plasmid pRhoKGyrB (which constitutively expresses the *gyrB* gene from the *neo* promoter). The presence of plasmid pRhoKGyrB enabled the strains DW5 and ∆RC6 to grow in the presence of Nb at 40 µg/mL, which completely inhibited the growth of these strains lacking the plasmid (Figure S5). Therefore, an increase in *gyrB* expression results in an increase in resistance to Nb, consistent with GyrB being the target of Nb.

**Figure 5:**
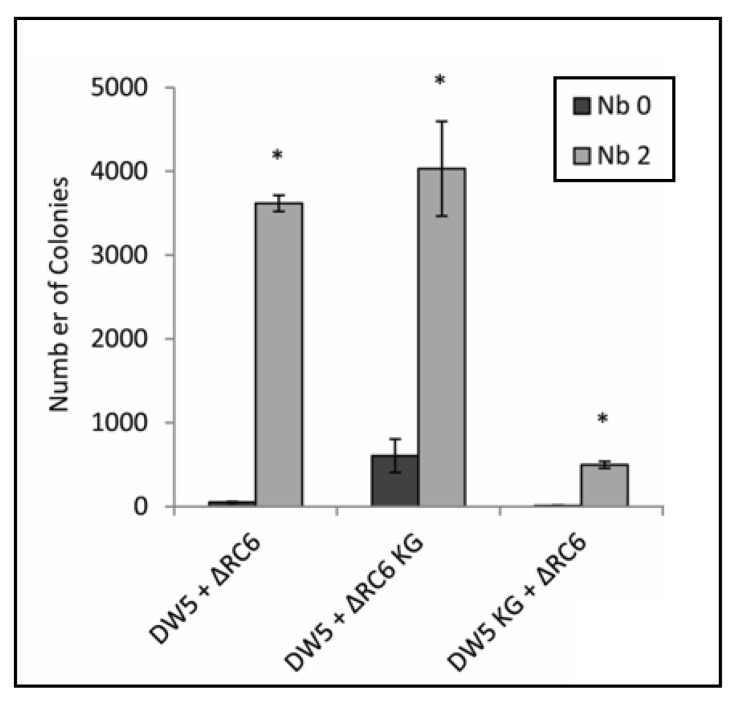
Transduction assays with NbR strains. Strains DW5, ∆RC6, DW5 containing plasmid pRhoKGyrB (DW5 KG), and ∆RC6 containing pRhoKGyrB (∆RC6 KG) were used. All combinations were grown with or without Nb at 2 µg/mL. Error bars indicate the standard deviation (n = 3), and * indicates *p* < 0.01 compared to the same mixture of strains grown without Nb.

When strain ∆RC6 containing plasmid pRhoKGyrB (designated as strain ∆RC6 KG in Figure 5) was grown in co-culture with strain DW5, the presence of Nb stimulated gene transduction, as it did in co-cultures of strains DW5 and ∆RC6. However, when strain DW5 contained plasmid pRhoKGyrB (designated as strain DW5 KG in Figure 5), there was a great decrease in the number of colonies obtained in the presence of Nb, compared to cultures in which neither strain or only ∆RC6 contained the plasmid. As in earlier experiments, all transductants examined (35 for each condition) resulted from gene transfer from ∆RC6 to DW5. Therefore, the increase in *gyrB* expression negated the stimulatory effect of Nb on gene transfer, but only when *gyrB* was overexpressed in the recipient strain. We interpret these data to mean that binding of Nb to GyrB stimulates gene transfer, and does so by increasing the frequency of allele replacement in the recipient cell.

## 4. Discussion

In this research, we demonstrated that subinhibitory levels of DNA gyrase inhibitors cause an increase in RcGTA-dependent gene transfer frequency in *R. capsulatus.* The gyrase inhibitors that showed this effect were Nb and Cb, both aminocoumarins that affect the B subunit of gyrase, and Cip, a fluoroquinolone that targets the A subunit of gyrase [20,21]. The effect of Nb on gene transfer was shown not to be due to an increase in production of RcGTA, on the basis of bioassays and Western blots probed with capsid antiserum (Figure 3 and Figure S3). This was unexpected, as antibiotic-induced transduction by prophages is typically due to increased phage induction [16,39,40]. Additionally, the non-homologous GTA found in *B. hyodysenteriae*, VSH-1, mediates increased gene transfer in the presence of subinhibitory levels of carbadox and metronidazole through increased transcription of VSH-1 genes [17]. Both carbadox and metronidazole are metabolized to products that interact with bacterial DNA and cause mutations and DNA strand breaks. The gyrase inhibitors studied here (Nb, Cb and Cip) also cause DNA damage, although they do this through stoppage of the DNA replication fork and deregulation of DNA supercoiling [41]. Therefore, although a spirochaete and an alphaproteobacterium both show an increase in gene transfer by non-homologous GTAs in response to DNA damaging agents, the mechanism by which the increase in gene transfer occurs is different.

To confirm that the effect of Nb on RcGTA-mediated gene transfer was manifested through DNA gyrase, Nb resistance (Nb^R^) was conferred on strains DW5 and ∆RC6 by constitutive expression of *gyrB* from the plasmid pRhoKGyrB (Figure 5 and Figure S4). When ∆RC6 (the RcGTA producer in this assay) overexpressed *gyrB*, there was little change in gene transfer frequency compared to the control cultures in which neither strain contained the plasmid. This result is consistent with the idea that Nb increases gene transfer frequency by an effect on the recipient cell, but not on the production of RcGTA. When DW5 (the recipient of RcGTA-borne genes in this assay) overexpressed *gyrB* in the presence of Nb, there was a much lower frequency of gene transfer, compared to the cultures in which neither strain or only ∆RC6 contained the plasmid. Because DNA gyrase is a heterotetramer of 2GyrA + 2GyrB and *gyrA* was not overexpressed it is likely that the excess in GyrB resulted in a mixture of heterotetramers in which one, both, or neither GyrB bound Nb, resulting in a mixed population of holoenzyme activities. Regardless, it appears that an excess of GyrB suppressed gene transfer resulting from a subinhibitory concentration of Nb, consistent with the idea that the Nb mode of action on transduction frequency is to increase recipient capability by inhibition of DNA gyrase activity. We consider in the following paragraphs two general ways in which inhibition of DNA gyrase activity could increase recipient capability.

It has been shown in a variety of in vivo and in vitro assays that DNA gyrase-dependent negative supercoiling is essential for homologous recombination [42]. This is true for recombination between covalently closed double-stranded DNA molecules as well as between a supercoiled molecule and linear DNA, which is the case for recombination between the linear RcGTA-borne DNA and the genome of the recipient cell. Therefore, because Nb inhibits DNA gyrase and DNA gyrase is needed for homologous recombination, whereas we found that Nb stimulated recipient capability that requires homologous recombination, the effect described here appears to arise from another process.

DNA gyrase-dependent DNA supercoiling density also affects gene transcription [43]. In one early study it was found that the expression of 7% of *E. coli* genes was affected by changes in the level of supercoiling, either by conditional mutation of the DNA gyrase gene *gyrB* or by the addition of a gyrase inhibitor such as Nb [44]. Therefore, Nb could in principle affect gene expression in *R. capsulatus* too, resulting in changes in the amounts of Com, DprA or RecA proteins needed for import and integration of RcGTA-borne genes [9,11,12]. On balance, we favor this possible mechanism to explain the stimulation of recipient capability by Nb, although we cannot specify whether transcription of any of the genes known to be required for recipient capability, or possibly a new gene that has not yet been identified, is affected by Nb.

Genome-wide expression studies would be useful in determining exactly how Nb influences the frequency of gene transfer. Additionally, elucidation of the exact mechanism of action of subinhibitory levels of Nb in *R. capsulatus* may benefit from Nb^R^ mutants that are resistant by a mechanism different from that of overexpression of GyrB. Understanding how gene transfer is affected in such mutants could clarify the role of DNA gyrase in RcGTA-mediated gene transfer.

The clinical and agricultural use of antibiotics has led to their widespread presence in natural environments at subinhibitory levels, as well as selection for resistance when levels are high [45]. Here, we show that transduction by RcGTA is influenced by subinhibitory concentrations of antibiotics that inhibit DNA gyrase. Given the prevalence of members of the *Rhodobacterales* and other alphaproteobacteria that contain RcGTA-like gene clusters in many ecosystems [46,47,48,49,50], gene transfer by RcGTA-like particles may be a significant player in alphaproteobacterial evolution. The presence of low levels of such antibiotics in the environment may change the rate of adaptation and evolution of these bacteria, because HGT provides a feedstock in addition to mutation upon which natural selection acts [51,52].

## Figures and Tables

**Table 1:** List of *R. capsulatus* strains.

Strain	Source	Description
B10	[25]	wild type
SB1003	[26]	RifR B10 derivative
ΔRC6	[27]	B10 background, *puf* operon knockout, KanR
DW5	[28]	SB1003 background, translationally in-frame *puhA* deletion
SBΔRC6	this study	transduction of ΔRC6 *puf* operon knockout into SB1003, KanR
ΔRC6_Δg4	this study	ΔRC6 with g4 (GTA protease gene) disruption, KanR/SpcR
DW5_Δg4	this study	DW5 with g4 (GTA protease gene) disruption, KanR
ΔLHII	[29]	SBI003 background, *puc* operon knockout, SpcR

## Supplementary Materials

The following supporting information can be downloaded at: https://www.mdpi.com/article/10.3390/genes13112071/s1, Figure S1: Schematic of transduction assay and bioassay; Figure S2: Kinetics of *R. capsulatus* WT strain SB1003 growth in the presence of subinhibitory concentrations of novobiocin; Figure S3: Transduction frequency between strains ΔLHII and ΔRC6; Figure S4: Western blot of transduction assay cultures probed with RcGTA capsid antiserum; Figure S5: Representative growth profile showing resistance to novobiocin by *gyrB* overexpression; Table S1. Variety of substances other than novobiocin, clorobiocin, and novobiocin tested in the transduction assay, using strains DW5 and ∆RC6.
