# Supplementary material for: DNA Gyrase Inhibitors Increase the Frequency of Bacteriophage-like RcGTA-Mediated Gene Transfer in Rhodobacter capsulatus
Source: Genes (Basel). 2022 Nov 9;13(11):2071. doi: 10.3390/genes13112071 (PMC9690577; doi:10.3390/genes13112071)
Supplement: Supplementary file 1 [file genes-13-02071-s001.zip › Supplementary Figs & Table_proof.pdf]

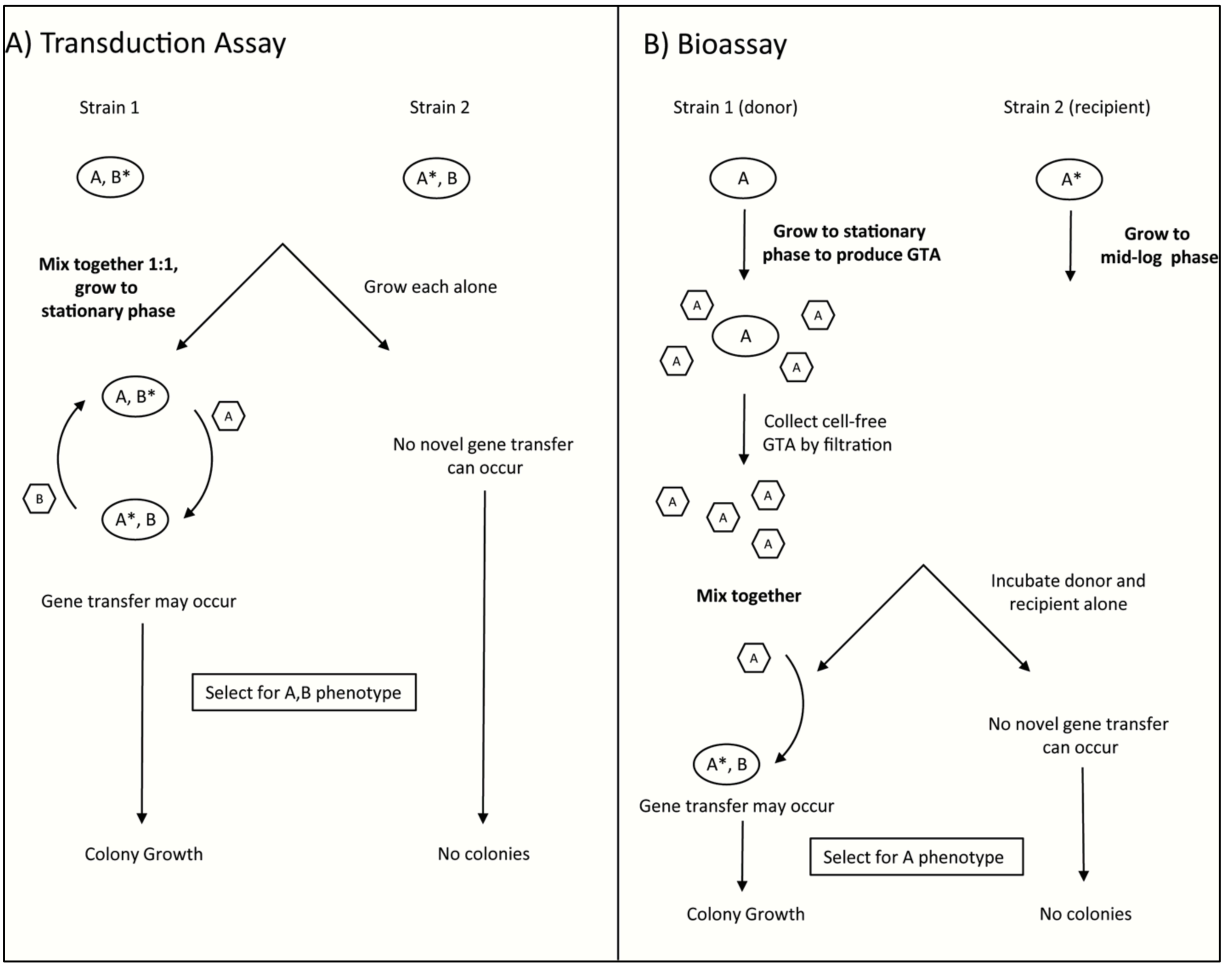

Figure S1. Schematic of transduction assay and bioassay. Antibiotics may be added to the steps in **bold** to determine their effect on the various stages of gene transfer.

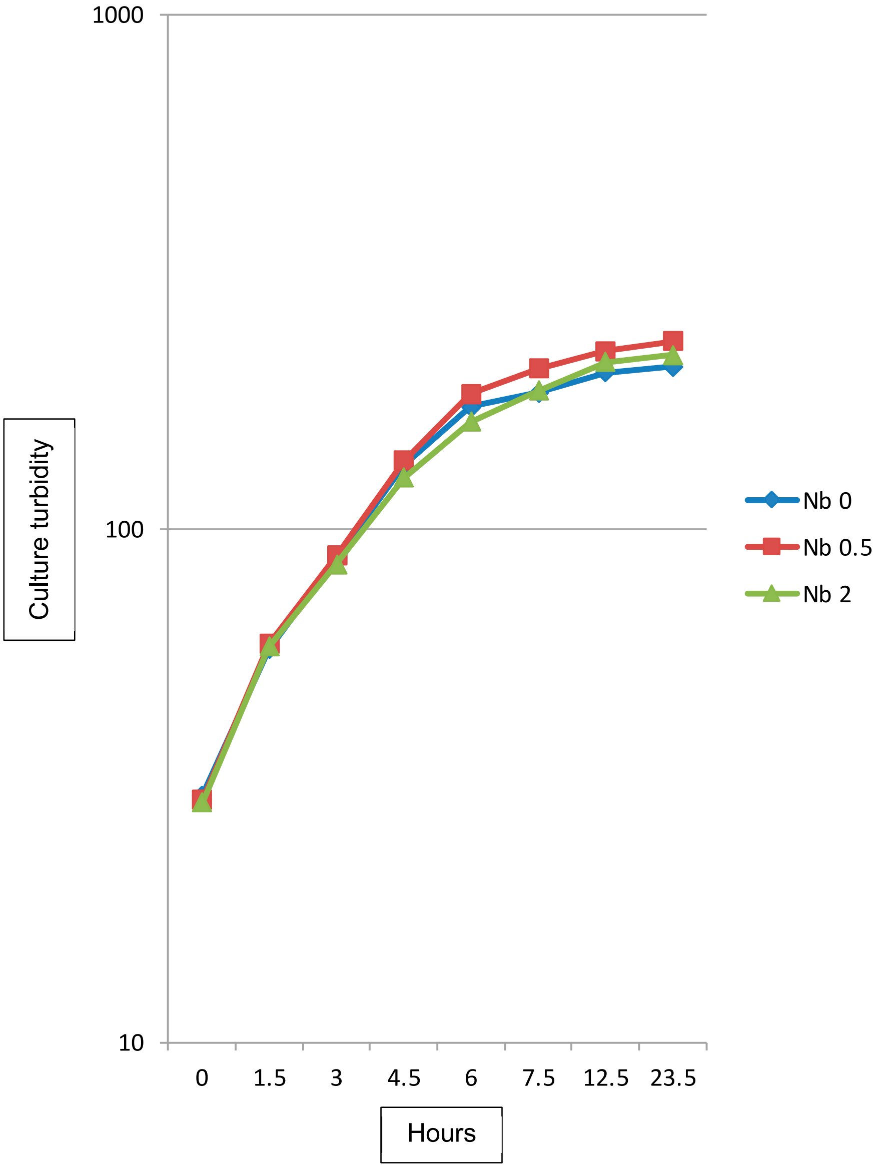

Figure S2. Kinetics of *R. capsulatus* WT strain SB1003 growth in the presence of subinhibitory concentrations of novobiocin (Nb). Concentrations given as µg/mL. Turbidity given as Klett photometer units

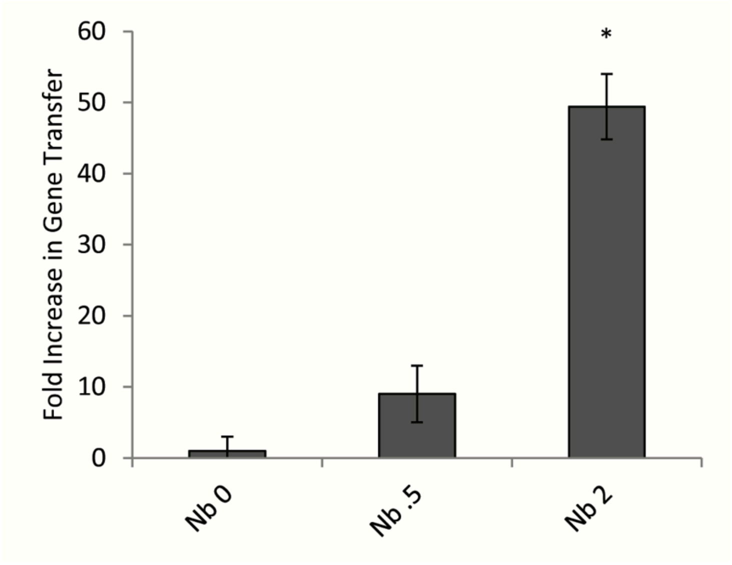

Figure S3. Transduction assay with alternative strains (ΔRC6 and ΔLHII instead of ΔRC6 and DW5) in the presence of Nb at 0, 0.5 or 2 μg/mL. Cultures were grown in triplicate. * $p < 0.005$ compared to cultures grown without antibiotic.

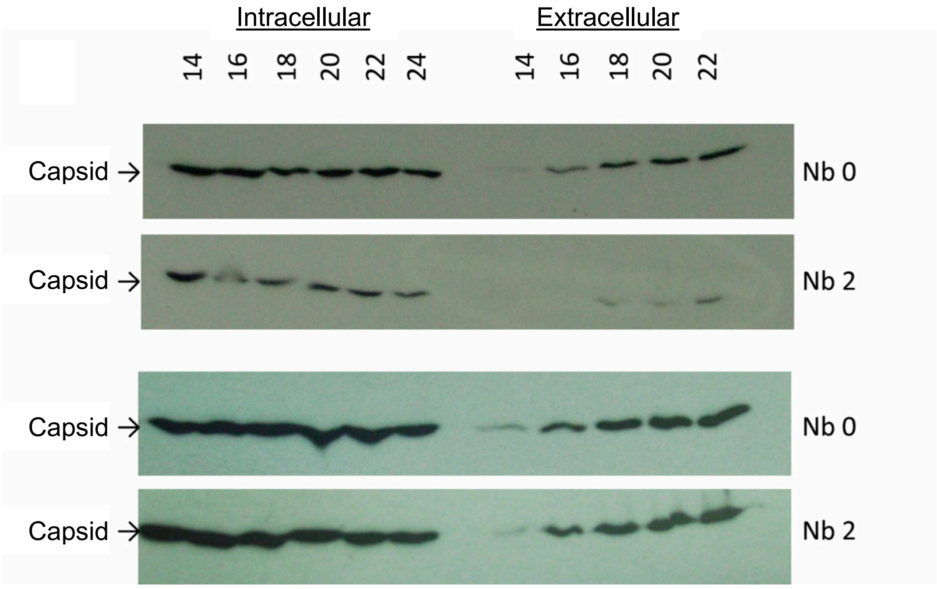

Figure S4. Western blot of transduction assay cultures probed with RcGTA capsid antiserum. Samples were removed from cultures at the hourly time points indicated above, cells (intracellular) were separated from culture medium (extracellular) by centrifugation, and equivalent culture volume amounts were loaded on SDS-PAGE gels. The amount of Nb in each culture is indicated on the right. The top two blots (Nb 0 and Nb 2) were developed in parallel and exposed to the same sheet of X-ray film, as were the bottom two blots.

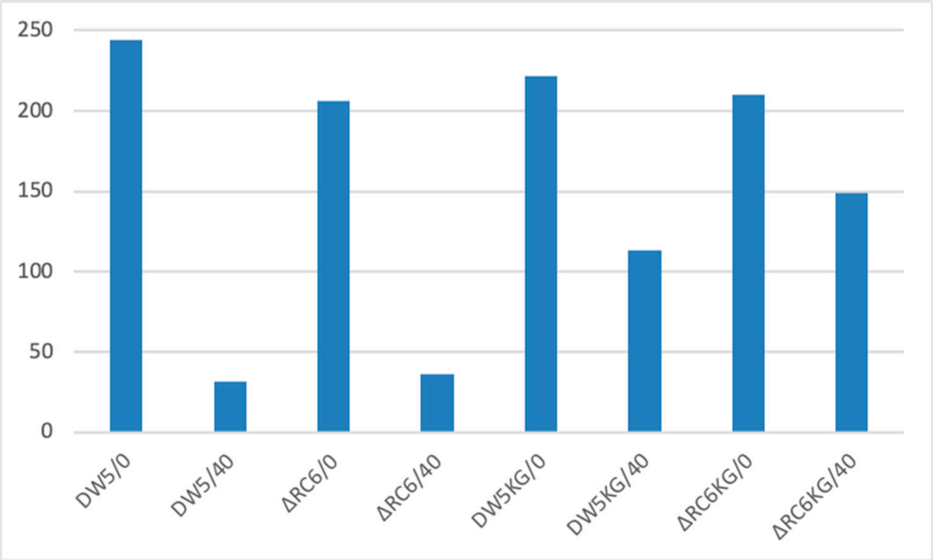

Figure S5. Representative growth profile showing resistance to novobiocin by *gyrB* overexpression. Culture turbidity in Klett units is given on the vertical axis (100 = 3.7 x 10^8 cfu/mL) after 55 hours of aerobic growth in YPS medium in the presence or absence of novobiocin (Nb). Cultures were inoculated at 30 Klett units. Strain designations (DW5 or DRC6, containing or not the *gyrB* overexpression plasmid pRhoKGyrB) are indicated below the horizontal axis, followed by the concentration of Nb in the medium (0 or 40 µg/ml). The strain suffix KG indicates strains that contain the *gyrB* overexpression plasmid.

Table S1. Variety of substances other than novobiocin, clorobiocin, and novobiocin tested in the transduction assay, using strains DW5 and ΔRC6.

Antibiotic or other substance	Trial number	Concentration μg/mL or μM	Number of colonies (averages of numbers in parentheses)	Fold change
**Streptomycin**	1	0	69 (52, 76, 81)	
	1	.5	166 (178, 161, 159)	2.4
	2	0	440 (336, 394, 589)	
	2	.5	135 (133, 122, 151)	0.31
	3	0	140 (112, 243, 64)	
	3	.5	17 (13, 11, 26)	0.12
	4	0	87 (68, 86, 107)	
	4	.5	0.3 (0, 0, 1)	0
**Rifampicin**	1	0	69 (52, 76, 81)	
	1	1	319	4.6
	1	2	427	6.2
	2	0	440 (336, 394, 589)	
	2	2	122 (140, 138, 87)	0.28
	3	0	156 (90, 195, 182)	
	3	2	25 (19, 34, 21)	.16
	3	4	30 (34, 27, 28)	.19
**Erythromycin**	1	0	440 (336, 394, 589)	
	1	.5	64 (61, 72, 59)	.15
**Bacitracin**	1	0	11 (9, 13, 10)	
	1	.5	7	.64
	1	2	14	1.3
	1	5	11	1
	1	10	8	.72
**Trimethoprim**	1	0	11 (9, 13, 10)	
	1	.5	12	1.1
	1	2	9	.82
	1	5	6	.55
	1	10	5	.45
**Coumermycin**	1	0	11 (9, 13, 10)	
	1	.5	0	0
	1	2	0	0
	1	5	0	0
	1	10	0	0
**HSLs**	1	0	87 (68, 86, 107)	
**-C12**	1	2 μM	120 (90, 150)	1.4
**-C16C**	1	2 μM	61 (54, 68)	.70
**-C16**	1	2 μM	44 (30, 57)	.51
**-C18**	1	2 μM	118 (124, 111)	1.4
**Indole acetic acid**	1	0	46 (66, 64, 9)	
**(IAA)**	1	2	75	1.6
	1	5	105	2.3
	1	10	255	5.5

2	0	190 (233, 197, 141)	
2	10	410 (436, 411, 382)	2.2
